# Five-Year Retrospective Review of Acute Generalized Exanthematous Pustulosis

**DOI:** 10.1155/2015/260928

**Published:** 2015-12-10

**Authors:** Chitprapassorn Thienvibul, Vasanop Vachiramon, Kumutnart Chanprapaph

**Affiliations:** Division of Dermatology, Faculty of Medicine, Ramathibodi Hospital, Mahidol University, Bangkok 10400, Thailand

## Abstract

*Background.* Acute generalized exanthematous pustulosis (AGEP) is an acute pustular eruption characterized by widespread nonfollicular sterile pustules. The aim of this study is to characterize the etiology, clinical features, laboratory findings, management, and outcome of patients with AGEP in Asians.* Patient/Methods*. A retrospective analysis was performed on patient who presented with AGEP between August 2008 and November 2012 in a tertiary center in Thailand.* Results.* Nineteen patients with AGEP were included. AGEP was generally distributed in seventeen patients (89.5%) and localized in two (10.5%). Fever and neutrophilia occurred in 52.6% and 68.4%, respectively. Hepatitis was found up to 26.3%. The most common etiology was drugs (94.7%), comprising of antibiotics (73.6%), proton pump inhibitors (10.5%), nonsteroidal anti-inflammatory drugs (5.3%), and herbal medicine (5.3%). Beta-lactams were the most common causal drug, particularly carbapenems and cephalosporins. This is the first report of* Andrographis paniculata* as an offending agent for AGEP. We found no differences between various treatment regimens (topical corticosteroid, systemic corticosteroid, and supportive treatment) regarding the time from drug cessation to pustules resolution (*P* = 0.171).* Conclusions.* We have highlighted the presentation of AGEP among Asians. We found high association with systemic drugs. Carbapenems were one of the leading culprit drugs. Finally, a localized variant was observed.

## 1. Introduction

Acute generalized exanthematous pustulosis (AGEP) is a pustular reaction characterized by an abrupt onset of numerous nonfollicular sterile pustules, arising within an erythematous and edematous background. It is rare with the reported incidence of one to five cases per million people per year [1]. The etiology includes systemic drugs (>90% of cases reported) especially antibiotics such as penicillin and macrolides, hypersensitivity to mercury, virus, contact dermatitis, and spider bite [2]. In this study, we described the demographic data, the etiologies, clinical features, laboratory findings, and management of patients with AGEP diagnosed in a tertiary hospital in Thailand.

## 2. Methods

### 2.1. Study Population

The medical records of 19 patients diagnosed with AGEP treated at Ramathibodi Hospital, Mahidol University, Bangkok, Thailand, from August 2008 and November 2012 were retrospectively reviewed. Patients 18 years of age and above who had definite or probable diagnosis of AGEP (final score ≥ 5) according to the grading system proposed by the study group of the European study of Severe Cutaneous Adverse Reactions (EuroSCAR) (Table 1) and with available histological data at the time of diagnosis were included in the study [1]. Patients who had psoriasis or pustular psoriasis confirmed histologically were excluded from the study.

### 2.2. Statistical Analysis

Statistical analyses were performed using computer software (SPSS version 18; SPSS Inc., Chicago, IL). Categorical variables (e.g., gender, prior drug allergy, distribution, culprit drug, clinical findings, and laboratory abnormalities) are expressed in percentage. Continuous variables (e.g., drug-AGEP latent period and resolution time) are reported as median. To compare various treatment regimens in correlation to the time of disease regression, ANOVA test was used. A *P* value of <0.05 was considered statistically significant.

## 3. Results

From August 2008 and November 2012, 25 patients of AGEP were identified. Nineteen patients had definite or probable diagnosis of AGEP according to the EuroSCAR AGEP validation score (final score ≥ 5). Fourteen patients had scores consistent with definite diagnosis and 5 patients were classified as probable cases of AGEP. Six cases classified as possible AGEP were excluded. The details of all patients and their reported comorbidities are summarized in Table 2. Of the 19 cases, ten (52.6%) were female and nine (47.4%) were male. The age ranged from 19 to 84 years, mean age of 52 years. None of the patients had personal or family history of psoriasis.

**Figure 1:**
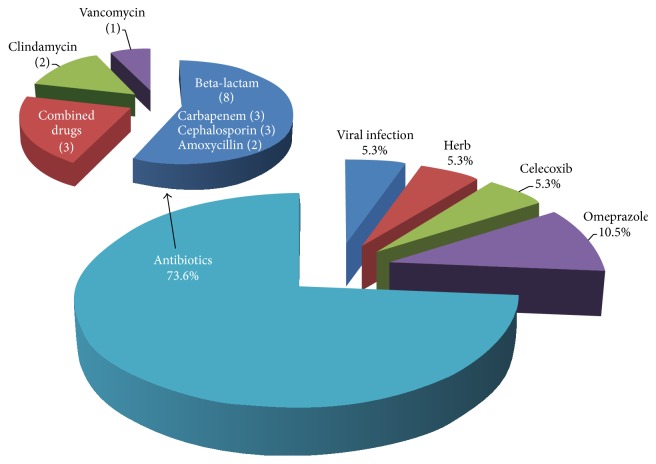
The etiology of acute generalized exanthematous pustulosis (AGEP) in this study.

Systemic drugs were the most common etiology. The criteria proposed by Naranjo et al. were used to identify the culprit drug [3]. The most frequent culprit was antibiotics in 14 patients (73.7%). Omeprazole was the offending drug in two patients (10.5%). Celecoxib and herbal drug, namely, the* Andrographis paniculata*, were the implicated medication in one patient. Among the culprit antibiotics, beta-lactams were the most common causal drug, particularly carbapenems and cephalosporins (3 patients each). One patient did not have history of drug exposure and had symptoms and signs suggesting viral infection such as fever, rhinorrhea, nasal congestion, cervical lymphadenopathy, myalgia, and arthralgia prior to skin eruption. The etiologies of AGEP in this study are shown in Figure 1. The latent period of drug administration before the onset of symptoms ranged from 1 hour to 25 days (median 3 days).

**Figure 2:**
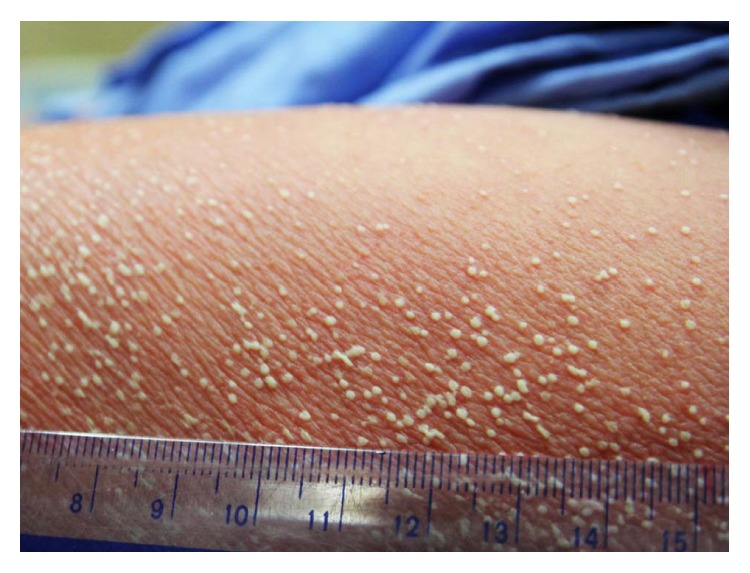
Nonfollicular, pinpoint, and superficial pustules on an erythematous background.

**Figure 3:**
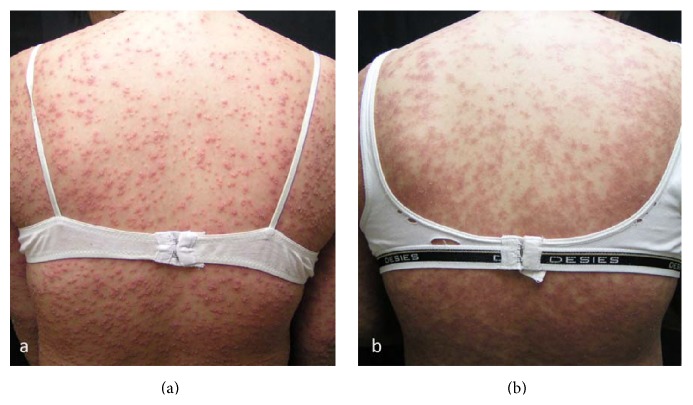
(a) Numerous small superficial pustules on erythematous base in a patient with AGEP. (b) Erythema and desquamation 3 days after discontinuation of culprit drug, and administration of systemic corticosteroid.

**Figure 4:**
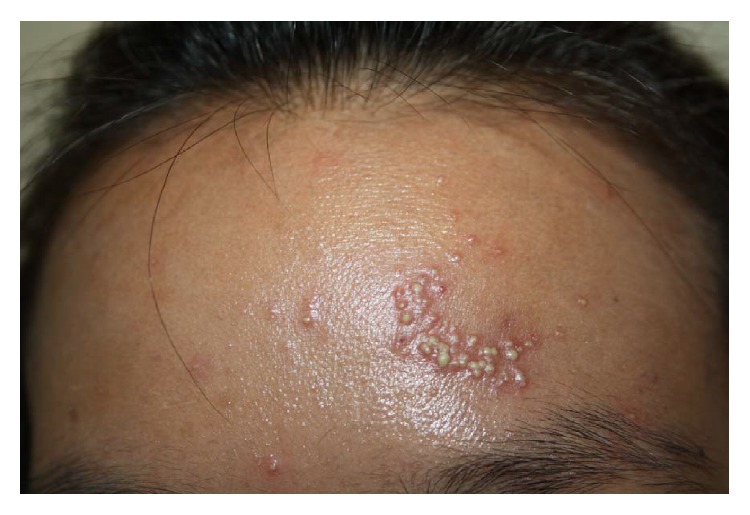
A 31-year-old female presented with ALEP on the face 1 day after taking herbal medicine.

All patients presented with nonfollicular, pinpoint, and superficial pustules on erythematous background (Figure 2), which were generally distributed in 17 of the patients (Figure 3(a)). The remaining 2 patients had localized lesions on the face (Figure 4) and the upper back, respectively. Fever was presented in 10 patients (52.6%). Facial edema was observed in 6 patients (31.6%). Three patients (15.8%) had at least one area (oral, ocular) of mucosal involvement. Pustules which coalesced to form pus-filled bullae resulted in cutaneous erosion in one patient. Resolution of pustules with desquamation (Figure 3(b)) was seen in 17 patients (89.5%). Cervical lymphadenopathy was observed in 2 patients.

Thirteen patients (68.4%) had associated leukocytosis (normal 4,000–10,000/*μ*L) and neutrophilia. Two patients (10.5%) had eosinophilia (eosinophil count ≥ 500/*μ*L or above 10% if the leukocyte count was lower than 4,000/*μ*L). Five patients (26.3%) had hepatocellular involvement (the highest level of serum aspartate aminotransferase and/or alanine aminotransferase exceeded two times the upper normal limit) which resolved later upon drug discontinuation and pustules resolution. None of the patients had other systemic involvement such as renal and pulmonary. Pus gram stain was performed in 12 patients and revealed numerous neutrophils without organism. Skin biopsy was carried out in every patient. All showed subcorneal pustules or intraepidermal pustules filled with neutrophils and some showed papillary dermal edema. Clinical characteristics and laboratory findings are summarized in Table 3.

Four patients had history of cutaneous adverse drug reaction. Three patients had recurrent AGEP and one had prior exanthematous drug eruption. Two patients had past history of beta-lactam-induced AGEP. The first patient previously had AGEP from penicillin and developed AGEP the second time from clindamycin. The other had the first and second episodes of AGEP from ampicillin and amoxicillin, respectively. One patient had multiple recurrent AGEP from numerous medications (amoxicillin, dicloxacillin, piroxicam, diclofenac, and omeprazole) and this time developed AGEP from omeprazole. Given that the same medication (omeprazole) was commenced in several events, proton pump inhibitor is believed to be the culprit drug on these occasions. One patient had maculopapular rash due to cinnarizine, acetazolamide, isoniazid, and rifampicin and developed AGEP this time from clindamycin.

Sixteen cases were hospitalized and 3 patients were seen at an outpatient department. Most cases (11 patients) were treated with topical corticosteroid. Six patients were given oral prednisolone, which were patients with extensive cutaneous diseases and/or systemic involvement such as hepatitis. The remaining 2 patients received supportive care. The median duration of drug cessation to resolution of pustules was 3 days (2–12 days). There was one patient who had vancomycin-induced AGEP with exceptionally long resolution time of 12 days. This patient has chronic kidney disease stage 4 which leads to longer drug clearance time.

There were no differences between various treatment regimens regarding the median duration of medication cessation to resolution of pustules which were 2 days for topical corticosteroid, 3 days for oral prednisolone, and 2.5 days for supportive care (*P* = 0.171). One patient had protected clinical course and developed generalized erythema and desquamation necessitating gradual tapering of systemic steroids. Another patient had erosions on the back which turned into ulcers and required further wound care.

## 4. Discussion

We present here the first case series of AGEP in Thailand. The most common etiology for AGEP was beta-lactams antibiotics. Among these, carbapenems has emerged as one of the leading causes of AGEP, reflecting the increasing use of broad-spectrum antibiotics. We also present the first report of a herbal drug, namely,* Andrographis paniculata*, to be the offending agent for AGEP.

Our study revealed no sexual predominance, in agreement with the previous two large series [4, 5]. A high association with systemic drugs was found in this study (94.7%), similar to a large report by Roujeau et al. (87%) [4]. However, this is in contrast to two series done in Korea and Taiwan which found lower association with systemic drugs, 63.8% and 62.5%, respectively [6].

Antibiotics were the leading cause of AGEP (73.7%), followed by omeprazole (10.5%), celecoxib (5.3%), and herbal drug (5.3%). Interestingly, a more recent antibiotic implicated for drug resistant nosocomial infection in the carbapenem group, such as imipenem and meropenem, was found to be the cause of AGEP in 3 patients (15.8%). This may reflect drugs commonly given in a tertiary care setting.

Herbal drug was the cause of AGEP in one patient.* Andrographis paniculata*, in the family Acanthaceae, or Fa-thalai-chon in the Thai language, is one of the most popular medicinal plants used in traditional medicine in various Asian countries. Its aerial parts, roots, and whole plant have been used for centuries for the treatment of various ailments such as fever, sore throat, cough, and stomachache and as antidiabetic and antioxidant agents [7, 8]. To the best of our knowledge, AGEP due to this herb has never been reported before. However, because it is commonly used in Asia, especially Thailand, and AGEP is a self-limiting disease, this particular side-effect might have been underestimated.

Viral infection was the suspected cause of AGEP in one patient based on the clinical presentation and peripheral blood picture profile; however, viral serology was not performed. Reports have shown that enterovirus, adenovirus, parvovirus B19, Epstein-Barr virus, cytomegalovirus, and hepatitis B virus were related to AGEP [9].

The pathophysiology of AGEP appears to be delayed type hypersensitivity to a specific drug. After exposure to the causative agent, activation of specific CD4 and CD8 occurs. These T cells then migrate to the skin and the drug-specific CD8 T cells use perforin/granzyme B and Fas ligand to cause keratinocytes apoptosis resulting in epidermal vesicle formation. Drug-specific T cells (mainly) and keratinocytes (to a lesser extent) produce CXCL8 (IL-8), a potent neutrophil-attracting chemokine, which leads to neutrophil accumulation within the vesicles. Moreover, interferon gamma and granulocyte/macrophage colony-stimulating factor are enhanced which help increase the survival of neutrophils and augment the formation of neutrophil accumulation [10, 11]. Peripheral blood of AGEP patients shows increase in circulating Th (T helper) 17, as Th17 produces IL-17 and IL-22, which have synergistic effect on the production of CXCL8 by keratinocytes and further direct neutrophil aggregation [11, 12]. In addition, systemic involvement in AGEP such as hepatitis may be due to circulating IL-17 and IL-22 [13]. IL-22 is detected in many inflammatory diseases [13]. However, the exact mechanism of AGEP remains uncertain. Patch test and lymphocytic transformation test are promising diagnostic tests for this type of drug hypersensitivity. Although not a part of the EuroSCAR validation score, it has been well known that patch testing could induce dermatologic reaction with corresponding drug and could be one of the features of AGEP [14, 15].

In this study, the median latent period between drug initiation and skin eruption was 3 days (1 hour–25 days). However, the interval of more than 10 days was found in 5 patients. Two of these patients received omeprazole and celecoxib, which were nonantibiotics. This supports data from a previous study that drugs other than antibiotics had longer time between the administration of suspected drug and the onset of skin eruption [4]. In general, the short interval between drug administration and the development of rash in AGEP is probably from prior sensitization and/or an immune recall phenomenon induced by T cell reactivation. Therefore, patients are more likely to have had a lifetime exposure to antibiotics or to drugs of similar structures than from nonantibiotics. In another patient where omeprazole was the suspected culprit, the drug was commenced 3 days before the development of AGEP; however, this patient had previous recurrent AGEP from many kinds of drugs including omeprazole. The pattern with a short interval again is probably due to previous sensitization. The remaining 3 patients that had long incubation period from the start of drug to the development of skin symptoms could result from primary sensitization.

Localized lesions of AGEP were found in two patients, one on the face and one on the upper back. Prange and colleagues first defined acute localized exanthematous pustulosis (ALEP) in 2005 [16]. They suggested that ALEP was a variant of AGEP both clinically and histopathologically, localized to the flexural areas, face, neck, or chest.

Other less common features such as facial edema, mucosal involvement, blister, and erosion, which were found in our series, have been described previously [2, 4, 9, 17, 18]. Resolution of pustules with desquamation was found in all patients except for patients with localized lesions.

Fever (52.6%) and neutrophilia (68.4%) were detected lower than previous studies [4, 9, 17]. Eosinophilia was found in 10.5% corresponding to a previous report [9]. Hepatitis was found in 26.3% which was higher than prior reports [4, 6, 13]. Therefore, systemic involvement especially hepatitis should not be overlooked in patients with AGEP and withdrawal of the implicated drug is essential to avoid further hepatic injury.

History of prior cutaneous adverse drug reaction was detected in 4 patients (21.1%), comprising AGEP in 3 and maculopapular rash in 1. Recurrent AGEP is rarely reported in the literature. The major culprit drugs to cause recurrence are beta-lactam antibiotics, which is in consistence with an early report [19]. Herein, we also report another case with recurrent AGEP possibly from omeprazole.

The diagnosis of AGEP is determined on morphology, clinical presentation, laboratory data, and histopathology. Several diagnostic criteria have been proposed and a well-recognized scoring system elaborated by EuroSCAR project has been used in this study [1].

The differential diagnoses include generalized pustular psoriasis of Von Zumbusch, pustular vasculitis, pustular Sweet's syndrome, autoimmune blistering disease, and pustular eruption from infections such as bacterial folliculitis, dermatophyte, candida infection, herpes, and varicella infection. Although most can be excluded without difficulty, some may be a diagnostic challenge especially differentiating between AGEP and generalized pustular psoriasis [20].

Due to the self-limited and benign nature of this disease, treatment is usually not necessary except for symptomatic therapy and discontinuation of the offending drugs. However, in certain patients, the clinical course may be extensive and protracted necessitating systemic corticosteroids [17]. In this study, we found no differences between various treatment regimens (topical corticosteroid, systemic corticosteroid, and supportive treatment) regarding the duration of medication cessation to the resolution of pustules (*P* = 0.171). The clinical outcome was good in almost all patients. Only one patient had generalized erythema and desquamation requiring gradual tapering of systemic corticosteroid. Another patient with morbid-obesity had erosions on the back which ulcerated and required further wound care.

The limitations of this study are its retrospective nature and small number of subjects. Nevertheless, given the rarity of AGEP, large-scale studies are limited. Patch testing was not done as a part of the workup of AGEP.

## Figures and Tables

**Table 1:** Acute generalized exanthematous pustulosis validation score of the EuroSCAR study group [1].

Morphology	Score
Pustules	
Typical	2
Compatible	1
Insufficient	0
Erythema	
Typical	2
Compatible	1
Insufficient	0
Distribution/pattern	
Typical	2
Compatible	1
Insufficient	0
Pustular desquamation	
Yes	1
No/insufficient	0
Course	
Mucosal involvement	
Yes	−2
No	0
Acute onset (<10 d)	
Yes	0
No	−2
Resolution (<15 d)	
Yes	0
No	−4
Fever > 38°C	
Yes	1
No	0
Neutrophils > 7,000/mm	
Yes	1
No	0
Histology	
Other diseases	−10
Not representative/no histopathology	0
Exocytosis of neutrophils	1
Subcorneal and/or intraepidermal nonspongiform or unspecified pustule(s) with papillary edema or subcorneal and/or intraepidermal spongiform or unspecified pustules(s) without papillary edema	2
Spongiform subcorneal and/or intraepidermal pustule(s) with papillary edema	3

Interpretation: <0: no AGEP, 1–4: possible, 5–7: probable, and 8–12: definite.

**Table 2:** Patient data (demographics, underlying disease, drug exposure, onset of symptoms, EuroSCAR AGEP validation, and therapy).

Patient	Age (years)/sex	Drug allergy history	Comorbidities	Possible etiology and duration between drug initiation and AGEP	EuroSCAR score	Therapy
1	28/F	No	Cervical carcinoma stage IIIb	Omeprazole (15 days)	8	Topical steroid

2	84/M	No	Pulmonary tuberculosis, diverticular bleeding	Isoniazid, rifampicin, pyrazinamide, and ethambutol (16 days)	10	Supportive

3	73/M	No	COPD, pulmonary tuberculosis	Amoxicillin (3 days)	8	Topical steroid

4	38/F	Yes	Submucous myoma, hyperthyroidism	Clindamycin (1 day)	9	Supportive

5	74/F	No	Subarachnoid hemorrhage	Meropenem (4 days)	8	Oral prednisolone

6	48/F	No	Cervical carcinoma stage IIIb	Celecoxib (11 days)	7	Topical steroid

7	38/F	No	Ruptured appendicitis, Graves' disease	Ceftriaxone (2 days)	8	Topical steroid

8	65/M	No	Congestive heart failure	Piperacillin/tazobactam Levofloxacin (4 days)	7	Oral prednisolone

9	45/M	No	Accidental fingers amputation	Cefazolin (2 days)	8	Oral prednisolone

10	71/F	Yes	Rheumatoid arthritis, subacute lupus erythematosus, and lymph node tuberculosis	Clindamycin (2 days)	9	Topical steroid

11	53/M	No	Atypical mycobacterial infection	Amikacin, clarithromycin, levofloxacin, and imipenem (25 days)	9	Oral prednisolone

12	33/F	Yes	Morbid obesity post-Roux-en-Y gastrojejunostomy, carcinoma of the ovary	Omeprazole (3 days)	9	Topical steroid

13	68/F	No	Renal failure, old stroke, and sepsis with pancytopenia	Meropenem (1 hour)	8	Topical steroid

14	38/M	Yes	No	Amoxicillin (2 days)	9	Oral prednisolone

15	31/M	No	Upper respiratory tract infection	Herbal medicine (1 day)	6	Topical steroid

16	73/M	No	Chronic kidney disease with infected arteriovenous fistula	Vancomycin (21 days)	7	Topical steroid

17	76/M	No	Double-vessel disease admitting for coronary artery bypass graft	Imipenem (4 days)	6	Topical steroid

18	19/F	No	Donor for liver transplantation	Cefoxitin (3 days)	8	Topical steroid

19	36/M	No	No	Viral infection	9	Oral prednisolone

COPD, chronic obstructive pulmonary disease.

**Table 3:** Clinical characteristics and laboratory findings.

Pt.	Distribution	Fever	Facial edema	Oral involvement	Conjunctival involvement	Desquamation	Neutrophil count (/mL)	Eo	Hepatitis
1	Abdomen, arms, and ankles	−	−	−	−	+	4,602	−	−

2	Face, back, and upper chest	+	−	−	−	+	7,106	−	−

3	Face, axillae, and trunk	−	+	−	−	+	9,516	−	−

4	Face, trunk, arms, and legs	+	+	−	−	+	14,490	−	−

5	Abdomen, back	−	−	−	−	+	15,486	−	+

6	Face, trunk, arms, and legs	−	+	−	−	+	6,295	−	−

7	Trunk, hands, and feet	−	−	−	−	+	10,030	−	−

8	Neck, upper chest, and back	−	−	−	−	+	3,552	−	−

9	Axillae, groins, lateral trunk, inner thighs, and volar surfaces of arms	+	−	−	−	+	3,472	10% (WBC 5,260)	+

10	Proximal extremities	+	−	−	−	+	18,620	−	−

11	Chest, back, and intertriginous areas	+	−	−	−	+	13,975	−	+

12	Inframammary areas, lateral trunk, lower abdomen, and upper thighs	+	−	−	−	+	9,672	−	−

13	Inframammary areas, groins, and axillae	+	−	+	−	+	814	33% (WBC 1,480)	−

14	Face, trunk, and extremities (about 90% BSA)	+	+	−	+	+	11,025	−	+

15	Face	−	−	−	−	−	4,838	−	−

16	Face, neck, trunk, and proximal extremities	+	+	−	−	+	7,241	−	−

17	Upper back	−	−	−	−	−	9,316	−	−

18	Back, groins	−	−	−	−	+	11,398	−	−

19	Trunk, extremities	+	+	+	+	+	11,808	−	+

Remark: +, present; −, absent; Pt., patient; BSA, body surface area; WBC, white blood cells; Eo, eosinophils.
